# A Systematic Review of the Oral Health Status of Hemophilic Patients

**DOI:** 10.3390/children12040490

**Published:** 2025-04-10

**Authors:** Tatsuya Akitomo, Ami Kaneki, Chieko Mitsuhata, Ryota Nomura

**Affiliations:** Department of Pediatric Dentistry, Graduate School of Biomedical and Health Sciences, Hiroshima University, Hiroshima 734-8553, Japan; kaneki@hiroshima-u.ac.jp (A.K.); chiekom@hiroshima-u.ac.jp (C.M.); rnomura@hiroshima-u.ac.jp (R.N.)

**Keywords:** hemophilia, oral health status, systematic review

## Abstract

Background: Hemophilia is an inherited bleeding disorder, with the most well-known forms being hemophilia A and hemophilia B. It is important for patients with hemophilia to maintain good oral health and prevent oral diseases because of their increased propensity to bleed. Few large-scale studies exist on the oral health status of patients with hemophilia. Methods: In our search across three databases (Web of Science, Scopus, and PubMed), only 14 articles about the oral health status of hemophilic patients were extracted. Oral health status was classified into caries status, periodontal status, and oral hygiene status and compared with that of a healthy group. Results: In total, 13 of the 14 papers investigated the dental caries status of hemophilia patients, and the most common report was “no difference” compared to healthy subjects (7 papers, 53.8%), followed by “poor” (4 papers, 30.8%). Periodontal status was investigated in 6 papers, of which 4 (66.7%) reported that the status of hemophilia patients was “poor” compared to that of healthy controls. Oral hygiene status was investigated in 12 studies, with 7 studies (58.3%) reporting it to be “poor” in hemophilia patients compared to healthy controls, followed by 3 studies (25.0%) reporting “no difference”. Conclusions: Oral hygiene status is associated with other items, such as caries and periodontal disease, and it is paramount in maintaining good oral health in hemophilic patients. In addition, regular dental visits improve oral hygiene, resulting in the prevention of oral disease. It is important to increase awareness of this information among patients with hemophilia and for medical and dental professionals to cooperate to improve patients’ oral health status, with the aim of improving their quality of life.

## 1. Introduction

Hemophilia is an inherited bleeding disorder caused by low concentrations of specific coagulation factors. The most well-known deficiencies are those of factor VIII (hemophilia A) and factor IX (hemophilia B), both of which show X-linked inheritance [1]. The incidence of hemophilia A is approximately 1 in 5000 and that of hemophilia B is 1 in 25,000 live male births [2]. Hemophilia is suspected when a child presents with easy bruising, spontaneous bleeding (particularly into the joints, muscles, and soft tissues), or excessive bleeding following trauma or surgery. A definitive diagnosis depends on a factor assay to demonstrate a deficiency of factor VIII or IX [3].

Dental caries and periodontitis are common oral diseases that affect many people worldwide [4,5,6]. In recent years, scientific research in the medical field has shown that health starts from the mouth, and the importance of oral care has been drawing increasing attention [7]. Patients with hemophilia have an increased risk of significant bleeding from invasive dental procedures without appropriate pre-operative precautions or treatment [8]. Therefore, according to the World Federation of Hemophilia guidelines, people with hemophilia should maintain good oral health to prevent oral diseases and conditions such as dental caries, gingivitis, and periodontitis—which may cause serious gum bleeding, especially in those with severe/moderate hemophilia—and to avoid the need for major dental surgery [9,10,11]. Many studies have investigated the oral health status of hemophilic patients; however, the findings have varied, and the associations between oral health status and hemophilia remain unclear [12,13,14,15]. In addition, most dentists have either no or very limited experience in managing dental problems in hemophilic patients, and they may be reluctant to undertake required invasive procedures [8]. Having detailed information about the oral conditions of hemophilia patients would enable dental professionals to understand the conditions and provide appropriate advice and treatment. It also helps to reduce anxiety for dental professionals when dealing with patients with hemophilia. In addition, sharing this information with medical professionals leads to improvement of the knowledge of all health care professionals involved in hemophilia care, resulting in improvement of the quality of life of the patients.

From the above, we highlight the need for a large-scale survey on the oral health conditions of hemophilic patients to achieve optimal oral management. The aim of this systematic review is to clarify the oral health status of patients with hemophilia and provide medical and dental professionals with this information.

## 2. Materials and Methods

### 2.1. Search Strategy

The review protocol was developed in accordance with the Preferred Reporting Items for Systematic Reviews and Meta-Analyses (PRISMA) statement (see Supplementary File S1) [16]. A literature search of three databases (Web of Science, Scopus, and PubMed) was conducted by one of the authors on 23 December 2024. The articles were searched for manually using the terms “hemophilia” and “oral health status”.

### 2.2. Eligibility Criteria

All articles were selected based on the Patient, Intervention, Comparison, and Outcomes (PICO) framework as follows:

- (P) Patients with congenital bleeding disorders, including hemophilia;
- (I) None;
- (C) Healthy control without systemic disease;
- (O) Oral health status including dental caries, periodontal diseases, and oral hygiene.

The eligibility criteria for the systematic review are shown in Table 1. The inclusion criteria were determined as follows: articles that could be viewed in their entirety; articles with their full text in English; and clinical investigations compared with healthy controls that were not case reports or reviews. Articles that were not suitable for the objective of this review or used the wrong study design were excluded.

### 2.3. Study Selection

According to the inclusion and exclusion criteria, a literature analysis was performed by two independent examiners (T.A. and A.K.) to select the articles for this review. Decisions about contentious documents were resolved by discussion.

### 2.4. Data Extraction

The authors extracted the following information: title, authors, year, subject of study, evaluation items, results, and quick summary. The evaluation items related to oral health status were subdivided into “Caries status”, “Periodontal status”, and “Oral hygiene status”. Caries status included decayed, missing, and filled teeth (dmft and DMFT); periodontal status included the modified gingival index (MGI) and bleeding on probing (BOP) score, and so on; and oral hygiene status included the oral hygiene index (OHI) and plaque index (PI), and so on. In the quick summary, the oral health status of hemophilic patients was compared with that of healthy controls, with reference to descriptions in the literature and statistically significant differences, and a final evaluation was made using three categories: “good”, “no difference”, and “poor”.

### 2.5. Risk of Bias Assesment

A risk of bias assessment was conducted in accordance with the Cochrane Handbook for Systematic Reviews of Interventions [17]. In brief, each article was identified across 5 bias domains (selection bias, performance bias, detection bias, reporting bias, and attrition bias). The judgement of bias was either low risk, some concern, or high risk.

## 3. Results

**Figure 1:**
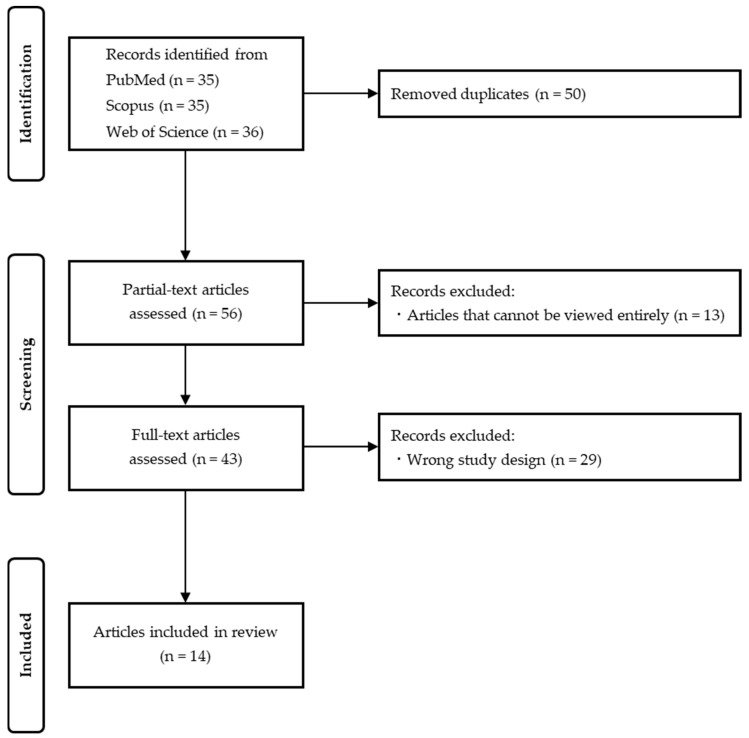
PRISMA flow diagram for literature search.

Figure 1 shows the PRISMA flow diagram for the literature search. Our literature search uncovered 106 articles across three databases (PubMed: 35, Scopus: 35, Web of Science: 36). Duplicates were removed and 13 articles were removed after partial-text article assessment to exclude articles that could not be viewed in their entirety. After a full-text article assessment of the 43 articles, 14 articles met each criterion and were included in this review. The subjects of the studies and the evaluation items are shown in Table 2, and the results and quick summaries are shown in Table 3 [18,19,20,21,22,23,24,25,26,27,28,29,30,31].

### 3.1. Comparison of the 14 Extracted Articles

Of the fourteen articles, four articles investigated all three items of caries status, periodontal status, and oral hygiene status; nine articles reported on two items; and one article reported on one item. Of the thirteen articles that investigated two or three items, four articles reported that all were “poor”; however, none reported that all were “good”.

### 3.2. Caries Status

Thirteen articles investigated the caries status of hemophilic patients. The most common answer was “no difference” in seven articles (53.8%), followed by “poor” in four articles (30.8%) and “good” in two articles (15.4%).

### 3.3. Periodontal Status

Periodontal status was the least common of the three evaluation items, featuring in only six articles. Of the six articles, four (66.7%) reported that the periodontal status of hemophilic patients was poorer than that of healthy controls. Only one article each (16.7%) reported the periodontal status as “good” or “no difference”.

### 3.4. Oral Hygiene Status

Twelve studies investigated oral hygiene status. Seven articles (58.3%) reported that the oral hygiene status of hemophilic patients was poorer than that of healthy controls, followed by “no difference” in three articles (25.0%) and “good” in two articles (16.7%).

### 3.5. Risk of Bias Assessment

The details of the risk of bias assessment are shown in Table 4. Regarding the risk of bias assessment, two papers were estimated as low risk in all five bias domains, and five papers were estimated as low risk in four bias domains. On the other hand, four papers were estimated to be high risk in at least one domain.

## 4. Discussion

Hemophilia is an inherited bleeding disorder, and it is important for those affected by it to maintain good oral health and prevent oral disease because of their increased propensity to bleed. However, there are few large-scale studies on the oral health status of hemophilic patients. We performed a literature search across three databases following PRISMA guidelines, and 14 articles matched the eligibility criteria and were extracted for this review. In reports regarding caries status, “no difference” compared to healthy controls was most common, while “poor” was most common for the periodontal status and oral hygiene status items.

### 4.1. Oral Health Status

Health starts in the mouth, and good oral health not only determines dental health, but is also a starting point for the general health and well-being of the body [7]. It is important for biting, chewing, smiling, speaking, and maintaining physiological well-being [32]. However, dental biofilms cause major oral diseases such as periodontitis and caries, which hamper oral hygiene [33]. Therefore, oral hygiene status is used to evaluate oral health status in addition to caries status and periodontal status [34,35,36]. Govindaraju et al. (2023) investigated the oral hygiene status of children with and without juvenile diabetes, and no significant differences were observed in the gingival status and the dental caries prevalence; however, the overall oral hygiene status was found to be poor in children with juvenile diabetes [37]. In addition, Mielnik-Błaszczak (1999) found that children with severe forms of hemophilia A and von Willebrand’s disease had worse dental status than other sick children. The author also described oral hygiene status as being worse in sick children than in healthy children [18]. This demonstrates how the oral condition can be assessed in detail by comparing the three items. In this study, we investigated the oral health status of hemophilic patients using these three items.

### 4.2. Caries Status of Hemophilic Patients

Of the articles that investigated the caries status of hemophilic patients, more than half reported no difference compared with the control group. Azhar et al. (2006), who reported that the caries status was poor in patients with hemophilia, concluded that oral and gingival bleeding led to reduced motivation to take care of the oral cavity [19]. Parents’ role in instilling in their children a positive attitude toward oral health and promoting their children’s oral health behavior at home is crucial [38,39]. Additionally, parents serve as important daily role models for their children; therefore, their oral health behavior is significantly correlated with their children’s oral health status [39,40,41]. Žaliūnienė et al. (2015) found good caries status in patients with hemophilia and concluded that toothbrushing techniques are learned well by children with hemophilia or by their parents [23]. Additionally, Salem et al. (2013) reported that if supportive care is provided for patients with hemophilia at a young age in a center for congenital bleeding disorders, including regular dental visits and regular education of patients and parents, good dental status is more likely to be achieved [21].

Many studies have confirmed a direct relationship between the intake of dietary sugars and dental caries across the life span, and diet plays a crucial role in caries disease [42,43]. Salem et al. (2013) pointed out that children with hemophilia have a high risk of caries during adolescence as a result of more frequent eating, more snack consumption, and less parental supervision [21]. These results suggest that although there is no difference in the risk of dental caries between hemophilic patients and healthy controls, the oral health of hemophilic patients may be worsened by poor motivation for oral care or dietary habits, just as in healthy people.

### 4.3. Periodontal Status of Hemophilic Patients

Two-thirds of the studies that included periodontal status reported that the periodontal status of patients with hemophilia was poor. Parvaie et al. (2020) concluded that a lack of awareness about proper brushing and a fear of bleeding were the cause of the poor periodontal status of patients with hemophilia [27]. Additionally, Acar et al. (2024) investigated the association between periodontal status indicators and OHI-S values, and found a strong association in the control group and a weak association in the hemophiliac group [31]. Debris or calculus accumulating on the tooth as a result of poor oral hygiene causes inflammation of the periodontium, affecting the periodontal status. However, the oral mucosa is affected by hemophilia, so even if the periodontium is healthy, it may tend to bleed even with mild stimulation, resulting in a poor periodontal score [31]. The BOP score and Community Periodontal Index (CPI) also use the presence or absence of bleeding as indicators [44,45]. Therefore, prolonged bleeding that occurs in patients with hemophilia may affect the evaluation.

Periodontal disease is classified as gingivitis or periodontitis. Gingivitis refers to inflammation of the gingiva due to the accumulation of bacteria and debris between the gum line and tooth, whereas periodontitis leads to loss of periodontal tissue attachment and then progresses to the loss of alveolar bone and loss of the affected teeth [46]. Ziebolz et al. (2011) evaluated periodontal status by observing periodontal bone loss on panoramic radiographs, and concluded that there were significant differences, although these differences were not clinically meaningful [20]. It may be advisable to use multiple different indices in combination when assessing the periodontal status of hemophilic patients. Studies that included periodontal status were the rarest among the reviewed articles. Additional research into the periodontal status of hemophilic patients, broken down by indicators, is needed. Regular dental checkups, including radiographic examinations, can help in the early detection of oral diseases. Medical and dental professionals should stress the importance of regular dental checkups. In addition, the standard method of periodontal therapy is scaling and root planning, and its efficacy is well documented by the demonstration of gains in clinical attachment levels, as well as reductions in probing pocket depths and bleeding on probing scores [47]. Further research is needed to investigate the effectiveness of periodontal therapy, including scaling and root planning, in hemophilic patients.

### 4.4. Oral Hygiene Status of Hemophilic Patients

More than half of the studies on oral hygiene status reported that the oral hygiene of hemophilic patients was poor, while only two (16.7%) reported that it was good. Kumar et al. (2018) reported significant differences in the toothbrushing habits of their study’s participants, which was reflected in the oral hygiene status of the two groups [24]. Gupta et al. (2022) reported that although the majority of the hemophilic patients participating in their study self-rated their oral health status as good, most had dental and periodontal disease, suggesting a low awareness of oral health [28]. Zaliuniene et al. (2014) suggests that congenital coagulation disorders are risk factors for oral diseases because patients with these disorders are afraid to use everyday prophylactic measures correctly to avoid bleeding episodes [48]. Providing appropriate information and motivation to patients may be important for improving poor oral hygiene. Othman et al. (2015) found that the multidisciplinary approach implemented by hematologists and other allied healthcare professionals acting as primary care providers together with the dental team resulted in good oral hygiene status in hemophilic patients [22]. Hemophilic children are less likely to be fearful than adults; they acquire fear after repeated hospital admissions and factor infusions. Although adults with hemophilia learn to live with the disease, they are more anxious about the possibility of bleeding episodes [24]. Patients and guardians should be provided with appropriate knowledge from an early age and medical and dental professionals should strive to work together to provide comprehensive treatment.

### 4.5. Dental Treatment of Hemophilic Patients

Particular care should be taken when patients with systemic diseases undergo dental procedures [49]. Hemophilia is mainly characterized by secondary hemorrhage following surgery/invasive procedures or trauma. Some cases require clotting factor replacement therapy, antifibrinolytic therapy, and local measures to obtain hemostasis perioperatively [50,51]. Therefore, it is important for hemophilic patients to maintain good oral health and prevent oral disease so as to avoid invasive dental procedures [9,10,11]. Consultation with a patient’s hematologist prior to invasive dental procedures should include discussions about plans for infusions of coagulation factors, blood products, and other measures prior to arrival at an outpatient, ambulatory, or office-based appointment [52]. It is important for dental professionals to cooperate with medical professionals on a day-to-day basis to ensure that appropriate dental treatment that is tailored to their condition is provided to hemophilic patients.

This review revealed that the oral health status of hemophilic patients was poor compared to that of healthy controls, particularly in terms of periodontal and oral hygiene status. On the other hand, for dental caries status, more than half of the studies reported no significant changes, suggesting that there is no causal relationship between hemophilia and dental caries. Oral hygiene status is associated with other items, such as caries and periodontal disease, and it is paramount in maintaining good oral health in hemophilic patients. Additionally, it was revealed that it is necessary to consider the characteristics of easy bleeding and to make comprehensive comparisons using multiple indicators to diagnose periodontal diseases. Dental professionals should understand this information and evaluate periodontal disease in hemophilia patients based on various test results. In addition, it is important that all health care professionals involved in hemophilia care share the information that oral hygiene status is the most important of the three topics and provide oral health education for hemophilic patients. On the other hand, the only limitation of this review is the short search phrases used to create the review. MeSH keywords and logical operators should be used to create a more detailed systematic review in the future.

## 5. Conclusions

Although there are few reports on the oral health status of hemophilic patients, a literature search yielded 14 articles that found that the oral health status of hemophilic patients is poorer than that of healthy controls. In particular, the proportion of “poor” ratings was higher for the items of periodontal status and oral hygiene status. This study highlights the need for medical and dental professionals to provide hemophilic patients with appropriate information about managing their oral hygiene to maintain optimal periodontal conditions. Further research and analysis in this field will contribute to improving the quality of life of hemophilic patients.

## Figures and Tables

**Table 1:** Eligibility criteria for systematic review.

Inclusion Criteria	Exclusion Criteria
Articles that could be viewed in their entirety.	Articles that were not suitable for the objective of this review.
Articles with their full text in English.	Articles that used the wrong study design.
Clinical investigations compared with healthy controls that were not case reports or reviews.	Case reports and reviews.

**Table 2:** Studies selected for review—subjects and evaluation items.

Author	Bleeding Diathesis Group	Control Group	Evaluation Items
Mielnik-Błaszczak M, 1999 [18]	Male patients aged 4–18 years (n = 80) Hemophilia A (n = 70) Hemophilia B (n = 7) von Willebrand’s disease (n = 3)	Male patients aged 4–18 years (n = 80)	Caries status Oral hygiene status
Azhar S, 2006 [19]	Hemophilic volunteers suffering from severe form of disease, i.e., clotting factor level < 2% and dependent on transfusions in case of surgery (n = 52) Average age was 16.6 (SD ± 3.2) years	Controls with age and gender matched (n = 192) Average age was 16.7 (SD ± 3.0) years	Caries status Peridontal status
Ziebolz D, 2011 [20]	Patients suffering from congenital coagulation disease (n = 15) Hemophilia A (n = 8) von Willebrand’s disease (n = 7) Average age was 39.2 (SD ± 8.3) years	Healthy patients receiving routine dental check-up were selected randomly as controls (n = 31) Average age was 36.4 (SD ± 9.6) years	Caries status Peridontal status Oral hygiene status
Salem K, 2013 [21]	Patients with congenital bleeding disorders aged 2–15 years (n = 46) Average age was 7.6 (SD ± 4.2) years	Children in same age and gender demographics were selected as control group (n = 46) Average age was 7.5 (SD ± 3.4) years	Caries status Oral hygiene status
Othman NA, 2015 [22]	Hemophilia patients aged 7–16 years with no systemic disease other than haemophilia (n = 50) Hemophilia A (n = 41) Hemophilia B (n = 8) Other type of Hemophilia (n = 1) Average age was 11.7 (SD ± 0.4) years	Control subjects were selected during oral health screening program and were matched in terms of age (n = 50) Average age was 12.0 (SD ± 0.2) years	Caries status Peridontal status Oral hygiene status
Žaliūnienė R, 2015 [23]	Patients 4 years or older listed in register of hemophilia patients (n = 76)	Control group was chosen from general population by randomly selecting subjects (n = 79)	Caries status Oral hygiene status
Kumar M, 2018 [24]	Patients diagnosed with hemophilia (n = 100) Hemophilia A (n = 86) Hemophilia B (n = 14) Average age was 20.0 years	Age-matched volunteers (n = 100) Average age was 20.1 years	Caries status Oral hygiene status
Kanjani V, 2020 [25]	Patients who had registered with Hemophilia Society (n = 50) Hemophilia A (n = 36) Hemophilia B (n = 14) Average age was 17.7 (SD ± 10.5) years	Healthy individuals matched with hemophilic individuals in terms of age and gender (n = 50) Average age was 17.7 (SD ± 10.5) years	Caries status Oral hygiene status
Kumar M, 2020 [26]	Patients diagnosed with hemophilia (A or B) who were registered with Hemophilia Society (n = 11) Average age was 19.4 years	Gender-matched healthy volunteers (n = 11) Average age was 19.5 years	Caries status Oral hygiene status
Parvaie P, 2020 [27]	Group selected by non-probability sampling method from patients with hemophilia referred to hemophilia center (n = 89) Hemophilia A (n = 73) Hemophilia B (n = 12) Other type of Hemophilia (n = 4) Average age was 26.6 (SD ± 14.8) years	Patients referred to dental clinic who were matched for age, sex (n = 89) Average age was 27.5 (SD ± 15.1) years	Periodontal status
Gupta U, 2022 [28]	Subjects with hemophilia aged 7 to 30 years (n = 300) Hemophilia A (n = 269) Hemophilia B (n = 31) Average age was 18.5 (SD ± 6.2) years	Controls recruited from among relatives who came along with hemophilic patients (n = 300) Average age was 19.2 (SD ± 6.1) years	Caries status Oral hygiene status
Czajkowska S, 2023 [29]	Patients, aged between 18 and 70 years, in whom congenital hemophilia A or B was diagnosed (n = 77) Hemophilia A (n = 64) Hemophilia B (n = 13) Mean age was 35 years	Control group consisted of healthy volunteers, matched according to age and gender (n = 50) Mean age was 29.5 years	Caries status Peridontal status Oral hygiene status
Sharma S, 2023 [30]	Young male individuals suffering from hemophilia and registered with Hemophilia Society (n = 200)	Young, healthy male individuals matched with case group with respect to age and gender (n = 200)	Caries status Oral hygiene status
Acar G, 2024 [31]	Patients diagnosed with severe hemophilia A or B (n = 48) Hemophilia A (n = 38) Hemophilia B (n = 10) Average age was 37.6 years	Control group of 49 individuals with same characteristics but without systemic diseases (n = 49) Average age was 42.0 years	Caries status Peridontal status Oral hygiene status

SD: standard deviation.

**Table 3:** Studies selected for review—results and quick summary.

Author	Results	Quick Summary Compared To Control Group		
		Caries Status	Periodondal Status	Oral Hygiene Status
Mielnik-Błaszczak M, 1999 [18]	No statistically significant differences were found in caries severity between the sick and healthy children. The value of OHI was significantly lower in in the sick group.	!	Not listed	−
Azhar S, 2006 [19]	The DMFT value was higher in the sick group. A high proportion of patients showed inflammation compared to controls when their periodontal disease was assessed using the MGI.	−	−	Not listed
Ziebolz D, 2011 [20]	The median DMFT values of patients and healthy controls were not significantly different. There was a statistically significant difference in periodontal bone loss, but the observed difference is not clinically meaningful. Patients had significantly better oral hygiene (modified Quigley–Hein Index).	!	!	+
Salem K, 2013 [21]	Patients were significantly more caries-free, with less decayed teeth in primary-permanent dentition. There was no significant difference in the value of OHI-S.	+	Not listed	!
Othman NA, 2015 [22]	No significant difference was found between haemophilia patients and controls for both primary (dft, dt, ft) and permanent (DMFT, DT, FT) teeth. The mean MGI for haemophilia patients was significantly lower than in controls. Although no significant difference was found in OHI-S, a significantly higher proportion of haemophilia patients had a better oral hygiene status compared to the controls.	!	+	+
Žaliūnienė R, 2015 [23]	In the deciduous dentition, the overall caries experience (dft) significantly statistically differed between the hemophilic patients (2.6 ± 2.6) and their matched healthy controls (6.1 ± 2.5). Although the mean and SD of dental plaque levels were higher in children with hemophilia, this difference was not statistically significant. On the other hand, hemophilic adults had significantly higher dental plaque levels compared to the control subjects.	+	Not listed	!
Kumar M, 2018 [24]	The mean dmft/DMFT scores were exactly the same for both the groups and not statistically significant. There was a statistically significant difference in OHI-S scores, with the hemophilic subjects exhibiting a poorer oral hygiene status when compared to the healthy group.	!	Not listed	−
Kanjani V, 2020 [25]	No significant distinction in DMFT was observed between the groups. When oral hygiene status was compared, a fair oral hygiene status was found in both hemophilic and healthy individuals.	!	Not listed	!
Kumar M, 2020 [26]	The DMFT score did not vary significantly between the groups. Higher OHI-S scores and a poor oral hygiene status were observed more in the hemophilia group than in the healthy controls.	!	Not listed	−
Parvaie P, 2020 [27]	Although the mean of the MGI and the Periodontal Index were higher in hemophilic patients than in healthy individuals, this difference was not statistically significant.	Not listed	−	Not listed
Gupta U, 2022 [28]	The caries prevalence was higher in hemophilic patients than in controls, and the DMFT score was significantly higher in those with hemophilia. The mean debris, calculus, and overall OHI score were significantly higher in those with hemophilia.	−	Not listed	−
Czajkowska S, 2023 [29]	The incidence of dental caries in patients with hemophilia was higher compared to that of healthy patients. The BOP score in hemophilia patients was higher, which shows a significant difference. A comparison regarding oral hygiene status based on the Approximal Plaque Index showed that the oral hygiene status of hemophilia patients was poor.	−	−	−
Sharma S, 2023 [30]	Hemophilic people had a considerably greater incidence of dental caries. In addition, their DMFT/DEFT and OHI-S scores were barely poorer than those of healthy people.	−	Not listed	−
Acar G, 2024 [31]	No significant difference was found between the patient and control groups in terms of the DMFT. The GI and gingival bleeding time index scores, which indicate the inflammatory response of the periodontium, were found to be significantly higher in the patient group with hemophilia than in the healthy control group. In addition, patients with hemophilia had significantly higher DI-S, CI-S, and OHI-S scores than those of the control group.	!	−	−

OHI: oral hygiene index, DMFT: decayed/missing/filled teeth in permanent dentition, OHI-S: simplified oral hygiene index, MGI: modified gingival index, dft: decayed/filled teeth in primary dentition, dt: decayed teeth in primary dentition, ft: filled teeth in primary dentition, DT: decayed teeth in permanent dentition, FT: filled teeth in permanent dentition, dft: decayed/filled teeth in primary dentition, SD: standard deviation, dmft: decayed/missing/filled teeth in primary dentition, BOP: bleeding on probing, DEFT: decaying extracted filled tooth, GI: gingival index, DI-S: simplified debris index, CI-S: simplified calculus index. + (green): good, ! (yellow): no difference, − (red): poor.

**Table 4:** Risk of bias assessment.

Study	Risk of Bias Domains				
	Selection Bias	Performance Bias	Detection Bias	Reporting Bias	Attrition Bias
Mielnik-Błaszczak M, 1999 [18]	!	+	+	+	+
Azhar S, 2006 [19]	!	!	−	+	+
Ziebolz D, 2011 [20]	−	+	+	+	+
Salem K, 2013 [21]	!	+	+	+	+
Othman NA, 2015 [22]	!	!	+	+	+
Žaliūnienė R, 2015 [23]	+	+	+	!	−
Kumar M, 2018 [24]	!	!	!	+	+
Kanjani V, 2020 [25]	!	!	!	+	+
Kumar M, 2020 [26]	−	!	!	+	+
Parvaie P, 2020 [27]	+	+	+	!	+
Gupta U, 2022 [28]	+	+	+	+	+
Czajkowska S, 2023 [29]	+	+	!	+	+
Sharma S, 2023 [30]	+	+	+	+	+
Acar G, 2024 [31]	!	+	!	+	+

+ (green): low risk, ! (yellow): some concerns, − (red): high risk.

## Data Availability

The data are available from the corresponding author upon reasonable request.

## Supplementary Materials

The following supporting information can be downloaded at: https://www.mdpi.com/article/10.3390/children12040490/s1, Supplementary File S1: PRISMA 2020 Checklist.

## References

[1] Bolton-Maggs P.H., Pasi K.J. (2003). Haemophilias A and B. Lancet.

[2] Nathwani A.C. (2022). Gene therapy for hemophilia. Hematol. Am. Soc. Hematol. Educ. Program.

[3] Srivastava A., Brewer A.K., Mauser-Bunschoten E.P., Key N.S., Kitchen S., Llinas A., Ludlam C.A., Mahlangu J.N., Mulder K., Poon M.C. (2013). Guidelines for the management of hemophilia. Haemophilia.

[4] Struzycka I. (2014). The oral microbiome in dental caries. Pol. J. Microbiol..

[5] Krishna R., De Stefano J.A. (2016). Ultrasonic vs. hand instrumentation in periodontal therapy: Clinical outcomes. Periodontol. 2000.

[6] Worthington H.V., MacDonald L., Poklepovic Pericic T., Sambunjak D., Johnson T.M., Imai P., Clarkson J.E. (2019). Home use of interdental cleaning devices, in addition to toothbrushing, for preventing and controlling periodontal diseases and dental caries. Cochrane Database Syst. Rev..

[7] Fiorillo L. (2019). Oral Health: The First Step to Well-Being. Medicina.

[8] Rajantie H., Alapulli H., Mäkipernaa A., Ranta S. (2013). Oral health care in children with haemophilia in Helsinki, Finland. Eur. Arch. Paediatr. Dent..

[9] Scully C., Diz Dios P., Giangrande P. (2008). Oral Care for People with Hemophilia or a Hereditary Bleeding Tendency.

[10] Kalsi H., Nanayakkara L., Pasi K.J., Bowles L., Hart D.P. (2012). Access to primary dental care for patients with inherited bleeding disorders. Haemophilia.

[11] Srivastava A., Santagostino E., Dougall A., Kitchen S., Sutherland M., Pipe S.W., Carcao M., Mahlangu J., Ragni M.V., Windyga J. (2020). WFH Guidelines for the Management of Hemophilia, 3rd edition. Haemophilia.

[12] Boyd D., Kinirons M. (1997). Dental caries experience of children with haemophilia in Northern Ireland. Int. J. Paediatr. Dent..

[13] Sonbol H., Pelargidou M., Lucas V.S., Gelbier M.J., Mason C., Roberts G.J. (2001). Dental health indices and caries-related microflora in children with severe haemophilia. Haemophilia.

[14] Evangelista L.M., Lima C.C., Idalino R.C., Lima M.D., Moura L.F. (2015). Oral health in children and adolescents with haemophilia. Haemophilia.

[15] Jangra B., Goswami M. (2017). Assessment of Dental Caries Experience and Periodontal Health Status among Children with Haemophilia in New Delhi, India—A Case Control Study. Oral Health Prev. Dent..

[16] Page M.J., McKenzie J.E., Bossuyt P.M., Boutron I., Hoffmann T.C., Mulrow C.D., Shamseer L., Tetzlaff J.M., Akl E.A., Brennan S.E. (2021). The PRISMA 2020 statement: An updated guideline for reporting systematic reviews. BMJ.

[17] Cochrane Training Cochrane Handbook for Systematic Reviews of Interventions. https://training.cochrane.org/handbook/current.

[18] Mielnik-Błaszczak M. (1999). Evaluation of dentition status and oral hygiene in Polish children and adolescents with congenital haemorrhagic diatheses. Int. J. Paediatr. Dent..

[19] Azhar S., Yazdanie N., Muhammad N. (2006). Periodontal status and IOTN interventions among young hemophiliacs. Haemophilia.

[20] Ziebolz D., Stühmer C., Hornecker E., Zapf A., Mausberg R.F., Chenot J.F. (2011). Oral health in adult patients with congenital coagulation disorders-a case control study. Haemophilia.

[21] Salem K., Eshghi P. (2013). Dental health and oral health-related quality of life in children with congenital bleeding disorders. Haemophilia.

[22] Othman N.A., Sockalingam S.N., Mahyuddin A. (2015). Oral health status in children and adolescents with haemophilia. Haemophilia.

[23] Žaliūnienė R., Aleksejūnienė J., Brukienė V., Pečiulienė V. (2015). Do hemophiliacs have a higher risk for dental caries than the general population?. Medicina.

[24] Kumar M., Pai K.M., Kurien A., Vineetha R. (2018). Oral hygiene and dentition status in children and adults with hemophilia: A case-control study. Spec Care Dent..

[25] Kanjani V., Annigeri R.G., Hanagavadi S., Manjunath M.R. (2020). Comparative analysis of oral health and treatment necessities in hemophilia individuals of Davangere population—A case control study. J. Family Med. Prim Care.

[26] Kumar M., Pai K.M., Vineetha R., Kurien A. (2020). Oral hygiene and dentition status in patients with congenital hemorrhagic disorders: A comparative study. Pesqui. Bras. Odontopediatria Clín. Integr..

[27] Parvaie P., Shaygan Majd H., Ziaee M., Sharifzadeh G., Osmani F. (2020). Evaluation of gum health status in hemophilia patients in Birjand (a case-control study). Am. J. Blood Res..

[28] Gupta U., Kumar A., Manjunath B.C., Aggarwal S., Singh A., Ahluwalia R. (2022). Comparative Evaluation of the Oral Hygiene Status and Prevalence of Dental Caries in Hemophiliac and non-Hemophiliac Patients. Cardiometry.

[29] Czajkowska S., Rupa-Matysek J., Gil L., Surdacka A. (2023). Assessment of Oral Health and Healthy Habits in Adult Patients with Congenital Hemophilia. Eur J. Dent..

[30] Sharma S., Shahi A.K., Chandra S., Abdul N.S., Singh B., Singh R., Shivakumar G.C. (2023). State of Dental Health and Management Needs of Young Hemophilic Patients: A Case-control Study. Int. J. Clin. Pediatr. Dent..

[31] Acar G., Aktaş A. (2024). Assessment of jaw bone mineral density, resorption rates, and oral health in patients with severe hemophilia: A case-control study. Acta Odontol. Scand..

[32] Furuta M., Yamashita Y. (2013). Oral Health and Swallowing Problems. Curr. Phys. Med. Rehabil. Rep..

[33] Müller L.K., Jungbauer G., Jungbauer R., Wolf M., Deschner J. (2021). Biofilm and Orthodontic Therapy. Monogr. Oral Sci..

[34] Andari S.E., Ghandour L.A., Chaaya M., Ghafari J.G. (2022). Oral health status in a Lebanese geriatric population. East Mediterr. Health J..

[35] Patel A.S., Jalihal S., Ankola A.V., Santhosh V.N., Ragu K., Thakker J., Coutinho D., Kabra L. (2024). Dental caries, oral hygiene status and deleterious habits among migrant construction workers of Belagavi, India. J. Prev. Med. Hyg..

[36] Banihashem Rad S.A., Esteves-Oliveira M., Maklennan A., Douglas G.V.A., Castiglia P., Campus G. (2024). Oral health inequalities in immigrant populations worldwide: A scoping review of dental caries and periodontal disease prevalence. BMC Public Health.

[37] Govindaraju L., Gurunathan D. (2023). Comparison of the Oral Hygiene Status in Children With and Without Juvenile Diabetes—A Comparative Study. Indian J. Dent. Res..

[38] de Castilho A.R.F., Mialhe F.L., de Barbosa T.S., Puppin-Rontani R.M. (2013). Influence of Family Environment on Children’s Oral Health: A Systematic Review. J. Pediatr. (Rio J.).

[39] Karamehmedovic E., Bajric E., Virtanen J.I. (2021). Oral Health Behaviour of Nine-Year-Old Children and Their Parents in Sarajevo. Int. J. Environ. Res. Public Health.

[40] Olak J., Nguyen M.S., Nguyen T.T., Nguyen B.B.T., Saag M. (2018). The Influence of Mothers’ Oral Health Behaviour and Perception Thereof on the Dental Health of Their Children. EPMA J..

[41] Arora A., Nargundkar S., Fahey P., Joshua H., John J.R. (2020). Social Determinants and Behavioural Factors Influencing Toothbrushing Frequency among Primary School Children in Rural Australian Community of Lithgow, New South Wales. BMC Res. Notes.

[42] Touger-Decker R., van Loveren C. (2003). Sugars and dental caries. Am. J. Clin. Nutr..

[43] Amato J.N., de Sousa Eskenazi E.M., Massaoka C., de Araújo de Assis C.R., Castelo P.M. (2023). Relation between caries experience and the consumption of sweetened drinks and processed food in children: A population-based study. Int. J. Dent. Hyg..

[44] World Health Organization (1997). Oral Health Survey: Basic Methods.

[45] Verrusio C., Iorio-Siciliano V., Blasi A., Leuci S., Adamo D., Nicolò M. (2018). The effect of orthodontic treatment on periodontal tissue inflammation: A systematic review. Quintessence Int..

[46] Gasner N.S., Schure R.S. (2025). Periodontal Disease. StatPearls [Internet].

[47] Solomon S.M., Timpu D., Forna D.A., Stefanache M.A., Martu S., Stoleriu S. (2016). AFM comparative study of root surface morphology after three methods of scaling. Mater. Plast..

[48] Zaliuniene R., Peciuliene V., Brukiene V., Aleksejuniene J. (2014). Hemophilia and oral health. Stomatologija.

[49] Thornhill M.H., Gibson T.B., Durkin M.J., Dayer M.J., Lockhart P.B., O′Gara P.T., Baddour L.M. (2020). Prescribing of antibiotic prophylaxis to prevent infective endocarditis. J. Am. Dent. Assoc..

[50] Farrkh A., Garrison E., Closmann J.J. (2016). Dental surgical management of the patient with hemophilia. Gen. Dent..

[51] Raso S., Napolitano M., Sirocchi D., Siragusa S., Hermans C. (2022). The important impact of dental care on haemostatic treatment burden in patients with mild haemophilia. Haemophilia.

[52] Hoang T., Dowdy R.A.E. (2024). Review of Inherited Coagulation Disorders. Anesth. Prog..

